# Dieth­yl(μ_3_-2-methyl-4-oxo-4*H*-pyran-3-olato-κ^4^ *O* ^3^,*O* ^4^:*O* ^3^:*O* ^3^)tris­(μ_2_-2-methyl-4-oxo-4*H*-pyran-3-olato-κ^3^ *O* ^3^,*O* ^4^:*O* ^3^)trizinc toluene disolvate

**DOI:** 10.1107/S1600536813010064

**Published:** 2013-04-20

**Authors:** Rafał Petrus, Joanna Petrus, Karolina Paszek, Piotr Sobota

**Affiliations:** a Faculty of Chemistry, University of Wroclaw, 50-383 Wroclaw, 14 F. Joliot-Curie, Poland

## Abstract

The title compound, [Zn_3_(C_2_H_5_)_2_(C_6_H_5_O_3_)_4_]·2C_7_H_8_, crystallizes with one complex mol­ecule solvated by two mol­ecules of toluene in the asymmetric unit. The Zn^II^ ions are coordinated by two terminal ethyl (Et) groups and four maltolate ligands, which act as μ_3_- and μ_2_-bridges. The metal atoms are arranged in an incomplete cubane Zn_3_O_4_ core structure, derived from one EtZnO_3_ tetra­hedron, one EtZnO_4_ bipyramid and one ZnO_6_ octa­hedron, sharing common corners. The structure is stabilized by weak C—H⋯O and C—H⋯π inter­actions.

## Related literature

For general background to zinc–maltolate complexes, see: Ahmed *et al.* (2000 ▶); Petrus & Sobota (2012*a* ▶,*b* ▶). For biological activity, see: Thompson *et al.* (2004 ▶, 2006 ▶). For ring-opening polymerization of cyclic esters, see: Chamberlain *et al.* (2001 ▶). For material chemistry, see: Boyle *et al.* (2004 ▶); Kaplunov *et al.* (2012 ▶). For incomplete cubane Zn_3_O_4_ core topology, see: Maxim *et al.* (2008 ▶); Romero *et al.* (2010 ▶). For the continuous shape measure, see: Alvarez *et al.* (2002 ▶).
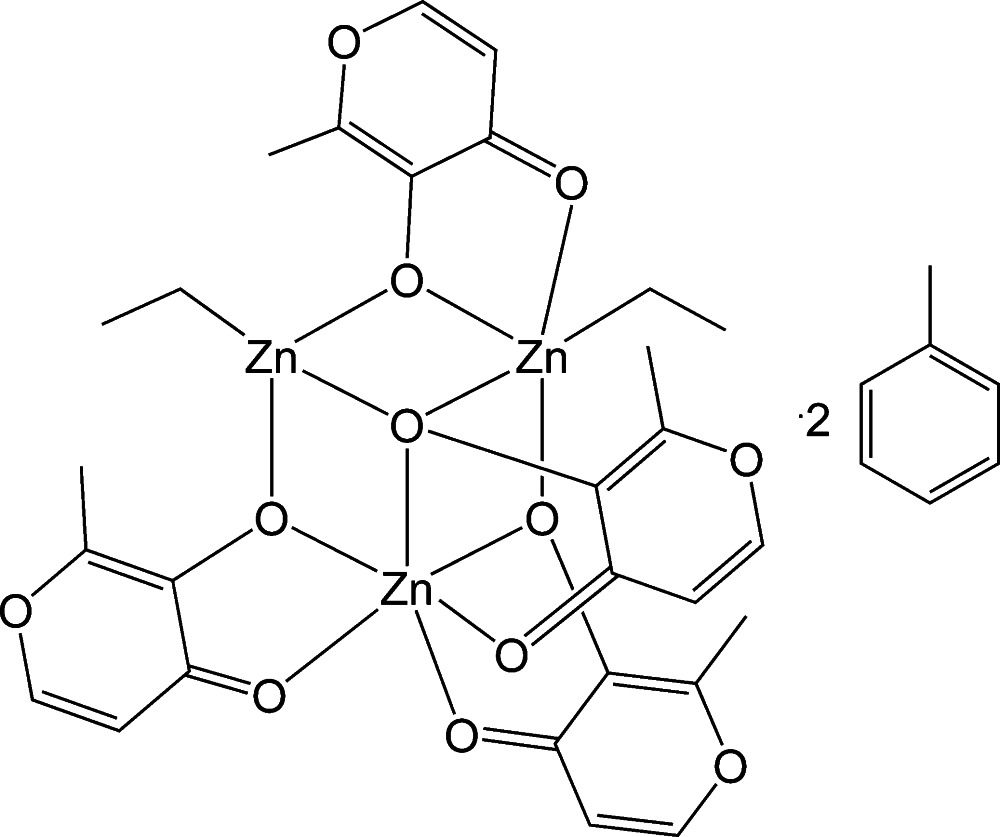


## Experimental

### 

#### Crystal data

[Zn_3_(C_2_H_5_)_2_(C_6_H_5_O_3_)_4_]·2C_7_H_8_
*M* *_r_* = 938.96
Monoclinic, 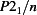

*a* = 10.125 (3) Å
*b* = 11.949 (4) Å
*c* = 34.653 (6) Å
β = 90.20 (2)°
*V* = 4192 (2) Å^3^
*Z* = 4
Mo *K*α radiation
μ = 1.76 mm^−1^
*T* = 100 K
0.43 × 0.37 × 0.09 mm

#### Data collection

KUMA KM4 CCD κ-geometry diffractometer
Absorption correction: analytical (*CrysAlis RED*; Oxford Diffraction, 2007 ▶) *T*_min_ = 0.580, *T*_max_ = 0.883
45983 measured reflections
9131 independent reflections
7444 reflections with *I* > 2σ(*I*)
*R*_int_ = 0.042

#### Refinement

*R*[*F* ^2^ > 2σ(*F* ^2^)] = 0.029
*wR*(*F* ^2^) = 0.066
*S* = 1.04
9131 reflections
521 parameters
H-atom parameters constrained
Δρ_max_ = 0.37 e Å^−3^
Δρ_min_ = −0.32 e Å^−3^

### 

Data collection: *CrysAlis CCD* (Oxford Diffraction, 2007 ▶); cell refinement: *CrysAlis RED* (Oxford Diffraction, 2007 ▶); data reduction: *CrysAlis RED*; program(s) used to solve structure: *SHELXS97* (Sheldrick, 2008 ▶); program(s) used to refine structure: *SHELXL97* (Sheldrick, 2008 ▶); molecular graphics: *OLEX2* (Dolomanov *et al.*, 2009 ▶); software used to prepare material for publication: *SHELXL97*.

## Supplementary Material

Crystal structure: contains datablock(s) global, I. DOI: 10.1107/S1600536813010064/aa2088sup1.cif

Structure factors: contains datablock(s) I. DOI: 10.1107/S1600536813010064/aa2088Isup2.hkl

Supplementary material file. DOI: 10.1107/S1600536813010064/aa2088Isup3.cdx

Additional supplementary materials:  crystallographic information; 3D view; checkCIF report

## Figures and Tables

**Table 1:** Selected bond lengths (Å)

Zn1—C1	1.971 (2)
Zn1—O3	2.0293 (14)
Zn1—O11	2.0077 (15)
Zn1—O17	2.0675 (14)
Zn2—C9	1.981 (2)
Zn2—O11	2.0943 (14)
Zn2—O12	2.1109 (15)
Zn2—O23	2.0193 (14)
Zn2—O3	2.6270 (15)
Zn3—O3	2.1060 (14)
Zn3—O4	2.0802 (15)
Zn3—O23	2.0759 (15)
Zn3—O24	2.0729 (14)
Zn3—O17	2.1439 (15)
Zn3—O18	2.1081 (15)

**Table 2:** Hydrogen-bond geometry (Å, °) *Cg*1 and *Cg*2 denote the centroids of the C29–C34 and C36–C41 rings, respectively.

*D*—H⋯*A*	*D*—H	H⋯*A*	*D*⋯*A*	*D*—H⋯*A*
C6—H6⋯*Cg*1^i^	0.95	2.53	3.477 (3)	172
C14—H14⋯O24^ii^	0.95	2.39	3.286 (3)	156
C20—H20⋯O17^iii^	0.95	2.40	3.332 (3)	166
C26—H26⋯O18^iv^	0.95	2.33	3.167 (3)	146
C28—H28*B*⋯*Cg*2^v^	0.98	2.72	3.411 (3)	128

Symmetry codes: (i) 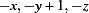; (ii) 
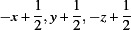
; (iii) 
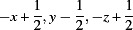
; (iv) 
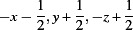
; (v) 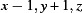.

**Fig. 1.**
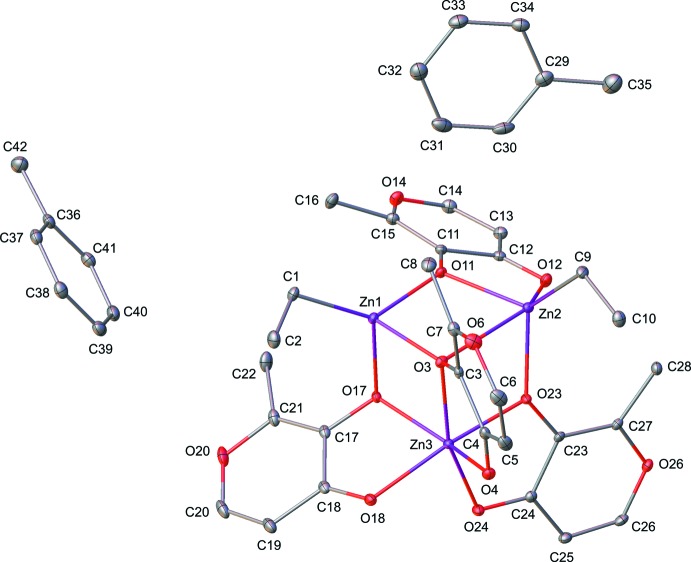
Molecular structure and atom-numbering scheme for (I), with displacement ellipsoids drawn at the 30% probability level. H atoms are omitted.

## supplementary crystallographic information

### Comment

In recent years alkoxo zinc complexes have attracted much attention for their use as initiators for the ring-opening polymerization of cyclic esters (Chamberlain *et al.*, 2001), metallotherapeutic drugs (Thompson *et al.*, 2006), and precursors of electroluminescent (Kaplunov *et al.*, 2012) and ceramic materials (Boyle *et al.*, 2004). In this contects have also been investigated so for related zinc–maltolate complexes (Ahmed *et al.*, 2000; Thompson *et al.*, 2004; Petrus & Sobota, 2012*a*, 2012*b*).

We describe here the solid state structure of zinc complex [Zn_3_(Et)_2_(MalO)_4_] (where MalOH = maltol), solvated by two molecules of toluene, (Fig. 1). The title zinc alkoxide has been previously reported in the monosolvated form [Zn_3_(Et)_2_(MalO)_4_]^.^C_7_H_8_, which crystallizes in the triclinic space group *P*1 (Petrus & Sobota, 2012*a*). The molecular structure comprises a trimer, containing incomplete cubane Zn_3_O_4_ core structure with different coordination modes around each zinc atoms (Maxim *et al.*, 2008; Romero *et al.*, 2010). The Zn1 ion is coordinated in a tetrahedral manner to one ethyl and two maltolate ligands in a monodentate coordination mode through atoms O11 and O17. The Zn2 atom is five-coordinated with EtZnO_4_ enviroment, comprise with zinc bonded ethyl group and three alkoxy (O3, O11, O23) and one carbonyl (O12) oxygen atoms from three maltolate ligands. The geometry around the Zn2 centre can be considered as distored trigonal bipyramid, as confirmed by the metric shape parameters S(Oh) = 3.59 (Alvarez *et al.*, 2002). The Zn3 ion is six-coordinated by three maltolate ligands in a bidentate chelating mode through the O atoms of alkoxy and carbonyl groups, forming five-membered Zn3/O3/C3/C4/O4, Zn3/O17/C17/C18/O18 and Zn3/O23/C23/C24/O24 chelate rings. The first of these metallacycles is twisted about the Zn3—O3 bond, while the rest have a conformation based on an envelope with the Zn3 atom as the envelope flap. The unsual elongated Zn2—O3 bond, *i.e.* by about 0.56 Å from the average Zn—O bond lengths within the Zn_2_O_2_ diamond cores, results in a high deformation of the central core geometry (Table 1). The title complex shows weak intermolecular C—H···O interactions between the trinuclear units and adjacent molecules to link them into extended chain (Table 2). The trinuclear cluster is also stabilized by C—H···π interactions with solvent molecules (Table 2).

### Experimental

The title compound was prepared by a procedure similar to that described in our earlier work (Petrus & Sobota, 2012*a*). Single crystals for XRD analysis were obtained from the concentrated mother liquor at room temperature. ^1^H NMR (C_6_D_6_, 500 MHz): δ 6.60 (4*H*, d, *J* = 5.1 Hz, CH, MalO), 6.02 (4*H*, d, *J* = 5.1 Hz, CH, MalO), 2.43 (s, 12H, CH_3_, MalO), 1.50 (6*H*, t, *J* = 7.9 Hz, CH_3_, Et), 0.61 (4*H*, k, *J* = 7.9, CH_2_, Et). ^13^C NMR (C_6_D_6_, 125 MHz): δ 178. 6 (4 C, C=O, MalO), 152.9 (4 C, CH, MalO), 152.2 - 149.9 (8 C, C—CH_3_, C—O, MalO), 111.5 (4 C, CH, MalO), 15.5 (4 C, CH_3_, MalO), 13.3 (2 C, CH_3_, Et), 0.1 (2 C, CH_2_, Et). FTIR-ATR (solid state): 2938 (w), 2921 (w), 2887 (w), 2851 (w), 1621 (*m*), 1614 (*m*), 1580 (*s*), 1536 (*m*), 1518 (*s*), 1457 (*m*), 1386 (w), 1363 (w), 1310 (w), 1287 (*m*), 1204 (*s*), 1234 (*m*), 1211 (*m*), 1191 (*s*), 1083 (w), 1042 (w), 984 (w), 946 (w), 852 (*s*), 832 (*s*), 763 (*m*), 719 (*m*), 708 (*m*), 607 (*m*), 591 (*m*), 586 (*m*), 546 (*s*), 529 (*s*), 510 (*m*), 499 (*m*), 484 (w), 406 (*m*). FTIR-ATR (toluene solution): 2940 (w), 2914 (w), 2890 (w), 2845 (w),1610 (*m*), 1589 (*s*), 1524 (*m*), 1495 (w), 1461 (*m*), 1390 (w), 1361 (w), 1281 (*s*), 1240 (w), 1202 (*m*), 1084 (w), 1040 (w), 922 (*m*), 852 (*m*), 830 (*m*), 762 (w), 590 (w), 544 (*m*), 463 (*s*), 440 (*m*), 409 (*m*), 401 (*m*).

### Refinement

Reported crystal showed weak reflections, especially at high diffraction angle, so these were omitted from the refinement for θ > 27°. All non-H atoms, were refined anisotropically. All H atoms were geometrically positioned and treated as riding on their parent atoms, with C—H = 0.95 Å for the aromatic, 0.98 Å for the methyl and 0.99 Å for the methylene with *U*_iso_(H) = 1.5 *U*_eq_(C) for methyl H atoms or 1.2 *U*_eq_(C) otherwise.

### Crystal data

**Table:**

[Zn_3_(C_2_H_5_)_2_(C_6_H_5_O_3_)_4_]·2C_7_H_8_	*F*(000) = 1936
*M**_r_* = 938.96	*D*_x_ = 1.488 Mg m^−^^3^
Monoclinic, *P*2_1_/*n*	Mo *K*α radiation, λ = 0.71073 Å
Hall symbol: -P 2yn	Cell parameters from 21726 reflections
*a* = 10.125 (3) Å	θ = 2.9–36.8°
*b* = 11.949 (4) Å	µ = 1.76 mm^−^^1^
*c* = 34.653 (6) Å	*T* = 100 K
β = 90.20 (2)°	Plate, colourless
*V* = 4192 (2) Å^3^	0.43 × 0.37 × 0.09 mm
*Z* = 4	

### Data collection

**Table:**

KUMA KM4 CCD κ-geometry diffractometer	9131 independent reflections
Radiation source: fine-focus sealed tube	7444 reflections with *I* > 2σ(*I*)
Graphite monochromator	*R*_int_ = 0.042
ω scans	θ_max_ = 27.0°, θ_min_ = 2.9°
Absorption correction: analytical (*CrysAlis RED*; Oxford Diffraction, 2007)	*h* = −12→10
*T*_min_ = 0.580, *T*_max_ = 0.883	*k* = −15→15
45983 measured reflections	*l* = −44→44

### Refinement

**Table:**

Refinement on *F*^2^	Primary atom site location: structure-invariant direct methods
Least-squares matrix: full	Secondary atom site location: difference Fourier map
*R*[*F*^2^ > 2σ(*F*^2^)] = 0.029	Hydrogen site location: inferred from neighbouring sites
*wR*(*F*^2^) = 0.066	H-atom parameters constrained
*S* = 1.04	*w* = 1/[σ^2^(*F*_o_^2^) + (0.034*P*)^2^ + 0.8469*P*] where *P* = (*F*_o_^2^ + 2*F*_c_^2^)/3
9131 reflections	(Δ/σ)_max_ = 0.001
521 parameters	Δρ_max_ = 0.37 e Å^−^^3^
0 restraints	Δρ_min_ = −0.32 e Å^−^^3^

### Special details

**Table:**

Geometry. All e.s.d.'s (except the e.s.d. in the dihedral angle between two l.s. planes) are estimated using the full covariance matrix. The cell e.s.d.'s are taken into account individually in the estimation of e.s.d.'s in distances, angles and torsion angles; correlations between e.s.d.'s in cell parameters are only used when they are defined by crystal symmetry. An approximate (isotropic) treatment of cell e.s.d.'s is used for estimating e.s.d.'s involving l.s. planes.
Refinement. Refinement of *F*^2^ against ALL reflections. The weighted *R*-factor *wR* and goodness of fit *S* are based on *F*^2^, conventional *R*-factors *R* are based on *F*, with *F* set to zero for negative *F*^2^. The threshold expression of *F*^2^ > σ(*F*^2^) is used only for calculating *R*-factors(gt) *etc*. and is not relevant to the choice of reflections for refinement. *R*-factors based on *F*^2^ are statistically about twice as large as those based on *F*, and *R*- factors based on ALL data will be even larger.

### Fractional atomic coordinates and isotropic or equivalent isotropic displacement parameters (Å^2^)

**Table:**

	*x*	*y*	*z*	*U*_iso_*/*U*_eq_	
Zn1	0.26829 (2)	0.439724 (18)	0.134257 (6)	0.01407 (6)	
Zn2	0.07663 (2)	0.667285 (19)	0.125066 (6)	0.01503 (6)	
Zn3	−0.00952 (2)	0.432645 (18)	0.174372 (6)	0.01362 (6)	
C1	0.3874 (2)	0.32296 (17)	0.11435 (6)	0.0209 (4)	
H1A	0.4283	0.3494	0.0901	0.025*	
H1B	0.4591	0.3096	0.1333	0.025*	
C2	0.3140 (2)	0.21269 (18)	0.10668 (7)	0.0285 (5)	
H2A	0.2808	0.1825	0.1311	0.043*	
H2B	0.3745	0.1587	0.0949	0.043*	
H2C	0.2396	0.2265	0.0891	0.043*	
O3	0.07472 (13)	0.44813 (11)	0.11915 (4)	0.0157 (3)	
C3	−0.00764 (19)	0.41008 (15)	0.09213 (6)	0.0152 (4)	
O4	−0.16934 (14)	0.38637 (12)	0.14003 (4)	0.0201 (3)	
C4	−0.1390 (2)	0.37953 (16)	0.10493 (6)	0.0179 (4)	
C5	−0.2278 (2)	0.34243 (18)	0.07548 (7)	0.0254 (5)	
H5	−0.3158	0.3223	0.0819	0.030*	
O6	−0.06397 (16)	0.36316 (13)	0.02767 (4)	0.0270 (4)	
C6	−0.1873 (2)	0.33606 (19)	0.03896 (7)	0.0293 (5)	
H6	−0.2483	0.3112	0.0199	0.035*	
C7	0.0255 (2)	0.40088 (17)	0.05440 (6)	0.0194 (4)	
C8	0.1551 (2)	0.4300 (2)	0.03700 (6)	0.0262 (5)	
H8A	0.1874	0.5002	0.0482	0.039*	
H8B	0.1446	0.4389	0.0090	0.039*	
H8C	0.2186	0.3700	0.0423	0.039*	
C9	−0.0003 (2)	0.71116 (18)	0.07464 (6)	0.0202 (4)	
H9A	0.0111	0.7930	0.0715	0.024*	
H9B	0.0515	0.6744	0.0540	0.024*	
C10	−0.1464 (2)	0.68336 (18)	0.06817 (7)	0.0251 (5)	
H10A	−0.1582	0.6020	0.0681	0.038*	
H10B	−0.1753	0.7140	0.0433	0.038*	
H10C	−0.1991	0.7163	0.0889	0.038*	
O11	0.26890 (13)	0.60774 (11)	0.13393 (4)	0.0150 (3)	
C11	0.34341 (19)	0.67853 (16)	0.15465 (5)	0.0146 (4)	
O12	0.15748 (14)	0.79574 (12)	0.15965 (4)	0.0202 (3)	
C12	0.2768 (2)	0.77899 (16)	0.16820 (6)	0.0165 (4)	
C13	0.3531 (2)	0.85483 (17)	0.19089 (6)	0.0199 (4)	
H13	0.3139	0.9210	0.2008	0.024*	
O14	0.54237 (14)	0.74190 (12)	0.18397 (4)	0.0212 (3)	
C14	0.4807 (2)	0.83225 (18)	0.19817 (6)	0.0219 (4)	
H14	0.5293	0.8824	0.2140	0.026*	
C15	0.4750 (2)	0.66578 (17)	0.16155 (6)	0.0186 (4)	
C16	0.5656 (2)	0.57722 (19)	0.14690 (7)	0.0253 (5)	
H16A	0.5142	0.5198	0.1333	0.038*	
H16B	0.6128	0.5430	0.1686	0.038*	
H16C	0.6295	0.6106	0.1291	0.038*	
O17	0.19558 (13)	0.42416 (11)	0.18968 (4)	0.0149 (3)	
C17	0.2217 (2)	0.32733 (17)	0.20767 (5)	0.0176 (4)	
O18	0.00872 (14)	0.26406 (11)	0.19152 (4)	0.0200 (3)	
C18	0.1169 (2)	0.24472 (17)	0.20831 (6)	0.0190 (4)	
C19	0.1442 (2)	0.14328 (18)	0.22914 (6)	0.0271 (5)	
H19	0.0790	0.0863	0.2307	0.033*	
O20	0.35898 (17)	0.20517 (13)	0.24421 (4)	0.0298 (4)	
C20	0.2613 (3)	0.12923 (19)	0.24636 (7)	0.0306 (5)	
H20	0.2761	0.0627	0.2607	0.037*	
C21	0.3390 (2)	0.30395 (17)	0.22475 (6)	0.0230 (5)	
C22	0.4570 (2)	0.3771 (2)	0.22506 (7)	0.0304 (5)	
H22A	0.4349	0.4493	0.2133	0.046*	
H22B	0.5280	0.3415	0.2103	0.046*	
H22C	0.4863	0.3889	0.2517	0.046*	
O23	−0.03074 (13)	0.60477 (11)	0.16908 (4)	0.0158 (3)	
C23	−0.10381 (18)	0.64703 (16)	0.19743 (5)	0.0141 (4)	
O24	−0.09670 (14)	0.46957 (12)	0.22689 (4)	0.0195 (3)	
C24	−0.13606 (19)	0.57043 (17)	0.22846 (6)	0.0161 (4)	
C25	−0.2127 (2)	0.61392 (18)	0.25957 (6)	0.0198 (4)	
H25	−0.2360	0.5672	0.2807	0.024*	
O26	−0.22396 (13)	0.79036 (11)	0.22951 (4)	0.0173 (3)	
C26	−0.2517 (2)	0.72161 (18)	0.25875 (6)	0.0206 (4)	
H26	−0.3010	0.7499	0.2799	0.025*	
C27	−0.15229 (18)	0.75339 (17)	0.19842 (6)	0.0149 (4)	
C28	−0.1401 (2)	0.84188 (17)	0.16854 (6)	0.0194 (4)	
H28A	−0.0715	0.8953	0.1763	0.029*	
H28B	−0.2247	0.8811	0.1658	0.029*	
H28C	−0.1162	0.8077	0.1438	0.029*	
C29	0.3482 (2)	0.8605 (2)	0.02648 (7)	0.0295 (5)	
C30	0.3451 (2)	0.7731 (2)	0.05304 (7)	0.0331 (6)	
H30	0.2861	0.7774	0.0743	0.040*	
C31	0.4249 (3)	0.6811 (2)	0.04938 (7)	0.0368 (6)	
H31	0.4203	0.6224	0.0678	0.044*	
C32	0.5119 (3)	0.6737 (2)	0.01900 (8)	0.0355 (6)	
H32	0.5677	0.6102	0.0164	0.043*	
C33	0.5172 (2)	0.7596 (2)	−0.00775 (7)	0.0314 (5)	
H33	0.5775	0.7551	−0.0286	0.038*	
C34	0.4361 (2)	0.8516 (2)	−0.00441 (7)	0.0282 (5)	
H34	0.4398	0.9093	−0.0232	0.034*	
C35	0.2573 (3)	0.9597 (2)	0.03019 (9)	0.0462 (7)	
H35A	0.1696	0.9399	0.0202	0.069*	
H35B	0.2928	1.0226	0.0154	0.069*	
H35C	0.2502	0.9810	0.0574	0.069*	
C36	0.8257 (2)	0.05574 (18)	0.10456 (7)	0.0250 (5)	
C37	0.7762 (2)	−0.02873 (18)	0.08076 (6)	0.0252 (5)	
H37	0.8321	−0.0612	0.0618	0.030*	
C38	0.6478 (2)	−0.06617 (19)	0.08413 (7)	0.0275 (5)	
H38	0.6160	−0.1240	0.0678	0.033*	
C39	0.5654 (2)	−0.01900 (19)	0.11148 (7)	0.0286 (5)	
H39	0.4772	−0.0448	0.1141	0.034*	
C40	0.6122 (2)	0.06595 (19)	0.13505 (7)	0.0289 (5)	
H40	0.5555	0.0988	0.1537	0.035*	
C41	0.7414 (2)	0.10325 (18)	0.13158 (7)	0.0269 (5)	
H41	0.7725	0.1618	0.1478	0.032*	
C42	0.9677 (2)	0.0922 (2)	0.10165 (8)	0.0359 (6)	
H42A	0.9839	0.1235	0.0759	0.054*	
H42B	1.0257	0.0277	0.1059	0.054*	
H42C	0.9860	0.1493	0.1213	0.054*	

### Atomic displacement parameters (Å^2^)

**Table:**

	*U*^11^	*U*^22^	*U*^33^	*U*^12^	*U*^13^	*U*^23^
Zn1	0.01236 (11)	0.01449 (11)	0.01537 (11)	0.00109 (9)	0.00032 (8)	−0.00205 (9)
Zn2	0.01327 (12)	0.01753 (11)	0.01428 (11)	−0.00031 (9)	−0.00046 (9)	0.00250 (9)
Zn3	0.01395 (12)	0.01278 (11)	0.01416 (11)	0.00048 (9)	0.00176 (9)	0.00036 (9)
C1	0.0158 (10)	0.0227 (10)	0.0240 (11)	0.0039 (8)	−0.0001 (8)	−0.0043 (9)
C2	0.0307 (13)	0.0210 (11)	0.0338 (13)	0.0052 (9)	−0.0031 (10)	−0.0048 (10)
O3	0.0131 (7)	0.0216 (7)	0.0124 (7)	−0.0006 (6)	−0.0025 (5)	−0.0028 (5)
C3	0.0160 (10)	0.0111 (9)	0.0186 (10)	0.0017 (7)	−0.0047 (8)	−0.0006 (7)
O4	0.0168 (7)	0.0197 (7)	0.0237 (8)	−0.0030 (6)	−0.0002 (6)	−0.0001 (6)
C4	0.0177 (10)	0.0131 (9)	0.0230 (11)	−0.0002 (8)	−0.0026 (8)	0.0014 (8)
C5	0.0210 (11)	0.0230 (11)	0.0320 (12)	−0.0063 (9)	−0.0102 (9)	0.0027 (9)
O6	0.0325 (9)	0.0313 (9)	0.0170 (7)	−0.0058 (7)	−0.0088 (7)	−0.0038 (6)
C6	0.0306 (13)	0.0288 (12)	0.0282 (12)	−0.0093 (10)	−0.0140 (10)	0.0015 (10)
C7	0.0220 (11)	0.0178 (10)	0.0183 (10)	0.0012 (8)	−0.0056 (8)	−0.0020 (8)
C8	0.0291 (12)	0.0324 (12)	0.0173 (10)	0.0035 (10)	0.0022 (9)	0.0002 (9)
C9	0.0198 (11)	0.0247 (11)	0.0160 (10)	0.0031 (9)	−0.0001 (8)	0.0042 (8)
C10	0.0257 (12)	0.0233 (11)	0.0264 (11)	0.0014 (9)	−0.0072 (9)	−0.0015 (9)
O11	0.0109 (7)	0.0157 (7)	0.0184 (7)	−0.0018 (5)	−0.0008 (5)	−0.0006 (6)
C11	0.0138 (10)	0.0153 (9)	0.0148 (9)	−0.0028 (7)	−0.0001 (7)	0.0014 (8)
O12	0.0147 (7)	0.0196 (7)	0.0261 (8)	0.0007 (6)	−0.0015 (6)	−0.0029 (6)
C12	0.0182 (11)	0.0171 (10)	0.0144 (10)	−0.0029 (8)	0.0020 (8)	0.0016 (8)
C13	0.0219 (11)	0.0185 (10)	0.0193 (10)	−0.0022 (8)	0.0018 (8)	−0.0032 (8)
O14	0.0150 (7)	0.0229 (7)	0.0256 (8)	−0.0036 (6)	−0.0036 (6)	−0.0056 (6)
C14	0.0241 (11)	0.0211 (10)	0.0205 (10)	−0.0059 (9)	−0.0029 (9)	−0.0043 (9)
C15	0.0183 (10)	0.0173 (10)	0.0203 (10)	−0.0039 (8)	−0.0008 (8)	−0.0011 (8)
C16	0.0147 (10)	0.0264 (11)	0.0347 (13)	0.0008 (9)	−0.0007 (9)	−0.0060 (10)
O17	0.0170 (7)	0.0138 (6)	0.0137 (6)	0.0034 (5)	−0.0004 (5)	0.0014 (5)
C17	0.0243 (11)	0.0174 (9)	0.0111 (9)	0.0057 (8)	0.0010 (8)	−0.0009 (8)
O18	0.0216 (8)	0.0155 (7)	0.0231 (8)	0.0007 (6)	0.0042 (6)	0.0015 (6)
C18	0.0260 (11)	0.0173 (10)	0.0137 (10)	0.0051 (8)	0.0047 (8)	−0.0006 (8)
C19	0.0399 (14)	0.0179 (11)	0.0235 (11)	0.0056 (10)	0.0046 (10)	0.0038 (9)
O20	0.0405 (10)	0.0263 (8)	0.0225 (8)	0.0156 (7)	−0.0096 (7)	0.0010 (7)
C20	0.0505 (16)	0.0174 (10)	0.0237 (12)	0.0106 (11)	−0.0001 (11)	0.0026 (9)
C21	0.0311 (12)	0.0201 (10)	0.0177 (10)	0.0083 (9)	−0.0054 (9)	−0.0027 (8)
C22	0.0270 (12)	0.0299 (12)	0.0342 (13)	0.0073 (10)	−0.0144 (10)	−0.0065 (10)
O23	0.0177 (7)	0.0149 (7)	0.0147 (7)	0.0028 (5)	0.0049 (6)	0.0015 (5)
C23	0.0090 (9)	0.0184 (10)	0.0150 (9)	−0.0008 (7)	0.0000 (7)	−0.0014 (8)
O24	0.0223 (8)	0.0193 (7)	0.0169 (7)	0.0030 (6)	0.0048 (6)	0.0028 (6)
C24	0.0132 (9)	0.0188 (10)	0.0163 (9)	−0.0014 (8)	−0.0018 (7)	−0.0006 (8)
C25	0.0182 (11)	0.0241 (11)	0.0171 (10)	−0.0003 (9)	0.0044 (8)	0.0015 (8)
O26	0.0150 (7)	0.0179 (7)	0.0190 (7)	0.0029 (6)	0.0010 (6)	−0.0044 (6)
C26	0.0150 (10)	0.0285 (11)	0.0183 (10)	0.0000 (9)	0.0052 (8)	−0.0026 (9)
C27	0.0105 (9)	0.0195 (10)	0.0148 (9)	0.0007 (7)	−0.0004 (7)	−0.0032 (8)
C28	0.0201 (11)	0.0172 (10)	0.0210 (10)	0.0038 (8)	0.0000 (8)	0.0010 (8)
C29	0.0227 (12)	0.0364 (13)	0.0294 (12)	−0.0093 (10)	−0.0032 (10)	−0.0091 (10)
C30	0.0246 (13)	0.0540 (16)	0.0207 (12)	−0.0184 (12)	0.0021 (10)	−0.0016 (11)
C31	0.0337 (14)	0.0446 (15)	0.0320 (13)	−0.0161 (12)	−0.0077 (11)	0.0113 (12)
C32	0.0314 (14)	0.0349 (14)	0.0402 (14)	−0.0051 (11)	−0.0056 (11)	−0.0021 (12)
C33	0.0289 (13)	0.0415 (14)	0.0239 (12)	−0.0087 (11)	0.0044 (10)	−0.0045 (10)
C34	0.0299 (13)	0.0310 (13)	0.0235 (12)	−0.0104 (10)	0.0000 (10)	−0.0002 (10)
C35	0.0398 (16)	0.0420 (16)	0.0568 (19)	−0.0031 (12)	0.0046 (14)	−0.0095 (14)
C36	0.0293 (12)	0.0181 (10)	0.0276 (12)	0.0028 (9)	−0.0015 (10)	0.0070 (9)
C37	0.0304 (13)	0.0219 (11)	0.0233 (11)	0.0063 (9)	−0.0013 (10)	0.0035 (9)
C38	0.0315 (13)	0.0234 (11)	0.0276 (12)	0.0011 (10)	−0.0095 (10)	0.0020 (10)
C39	0.0260 (13)	0.0274 (12)	0.0324 (13)	−0.0006 (10)	−0.0058 (10)	0.0094 (10)
C40	0.0330 (13)	0.0253 (11)	0.0284 (12)	0.0083 (10)	0.0023 (10)	0.0063 (10)
C41	0.0346 (13)	0.0185 (11)	0.0277 (12)	−0.0007 (9)	−0.0021 (10)	0.0022 (9)
C42	0.0327 (14)	0.0298 (13)	0.0451 (15)	−0.0042 (10)	0.0025 (12)	−0.0003 (11)

### Geometric parameters (Å, º)

**Table:**

Zn1—C1	1.971 (2)	C17—C21	1.355 (3)
Zn1—O3	2.0293 (14)	C17—C18	1.449 (3)
Zn1—O11	2.0077 (15)	O18—C18	1.260 (3)
Zn1—O17	2.0675 (14)	C18—C19	1.437 (3)
Zn2—C9	1.981 (2)	C19—C20	1.336 (4)
Zn2—O11	2.0943 (14)	C19—H19	0.9500
Zn2—O12	2.1109 (15)	O20—C20	1.345 (3)
Zn2—O23	2.0193 (14)	O20—C21	1.374 (3)
Zn2—O3	2.6270 (15)	C20—H20	0.9500
Zn3—O3	2.1060 (14)	C21—C22	1.480 (3)
Zn3—O4	2.0802 (15)	C22—H22A	0.9800
Zn3—O23	2.0759 (15)	C22—H22B	0.9800
Zn3—O24	2.0729 (14)	C22—H22C	0.9800
Zn3—O17	2.1439 (15)	O23—C23	1.331 (2)
Zn3—O18	2.1081 (15)	C23—C27	1.363 (3)
Zn1—Zn3	3.1430 (9)	C23—C24	1.450 (3)
C1—C2	1.536 (3)	O24—C24	1.271 (2)
C1—H1A	0.9900	C24—C25	1.428 (3)
C1—H1B	0.9900	C25—C26	1.346 (3)
C2—H2A	0.9800	C25—H25	0.9500
C2—H2B	0.9800	O26—C26	1.335 (2)
C2—H2C	0.9800	O26—C27	1.374 (2)
O3—C3	1.332 (2)	C26—H26	0.9500
C3—C7	1.355 (3)	C27—C28	1.486 (3)
C3—C4	1.450 (3)	C28—H28A	0.9800
O4—C4	1.258 (2)	C28—H28B	0.9800
C4—C5	1.428 (3)	C28—H28C	0.9800
C5—C6	1.334 (3)	C29—C30	1.392 (4)
C5—H5	0.9500	C29—C34	1.399 (3)
O6—C6	1.349 (3)	C29—C35	1.506 (4)
O6—C7	1.369 (2)	C30—C31	1.371 (4)
C6—H6	0.9500	C30—H30	0.9500
C7—C8	1.487 (3)	C31—C32	1.378 (4)
C8—H8A	0.9800	C31—H31	0.9500
C8—H8B	0.9800	C32—C33	1.384 (4)
C8—H8C	0.9800	C32—H32	0.9500
C9—C10	1.532 (3)	C33—C34	1.377 (4)
C9—H9A	0.9900	C33—H33	0.9500
C9—H9B	0.9900	C34—H34	0.9500
C10—H10A	0.9800	C35—H35A	0.9800
C10—H10B	0.9800	C35—H35B	0.9800
C10—H10C	0.9800	C35—H35C	0.9800
O11—C11	1.340 (2)	C36—C41	1.391 (3)
C11—C15	1.361 (3)	C36—C37	1.395 (3)
C11—C12	1.456 (3)	C36—C42	1.506 (3)
O12—C12	1.259 (2)	C37—C38	1.381 (3)
C12—C13	1.426 (3)	C37—H37	0.9500
C13—C14	1.342 (3)	C38—C39	1.384 (3)
C13—H13	0.9500	C38—H38	0.9500
O14—C14	1.342 (3)	C39—C40	1.385 (3)
O14—C15	1.376 (2)	C39—H39	0.9500
C14—H14	0.9500	C40—C41	1.387 (3)
C15—C16	1.491 (3)	C40—H40	0.9500
C16—H16A	0.9800	C41—H41	0.9500
C16—H16B	0.9800	C42—H42A	0.9800
C16—H16C	0.9800	C42—H42B	0.9800
O17—C17	1.340 (2)	C42—H42C	0.9800
			
C1—Zn1—O11	134.76 (8)	C15—C16—H16A	109.5
C1—Zn1—O3	122.44 (7)	C15—C16—H16B	109.5
O11—Zn1—O3	87.25 (5)	H16A—C16—H16B	109.5
C1—Zn1—O17	118.81 (8)	C15—C16—H16C	109.5
O11—Zn1—O17	95.53 (5)	H16A—C16—H16C	109.5
O3—Zn1—O17	84.11 (6)	H16B—C16—H16C	109.5
C1—Zn1—Zn3	133.28 (6)	C17—O17—Zn1	116.08 (11)
O11—Zn1—Zn3	91.84 (4)	C17—O17—Zn3	110.18 (12)
O3—Zn1—Zn3	41.45 (4)	Zn1—O17—Zn3	96.53 (6)
O17—Zn1—Zn3	42.66 (4)	O17—C17—C21	123.53 (19)
C9—Zn2—O23	123.58 (7)	O17—C17—C18	116.88 (18)
C9—Zn2—O11	125.55 (7)	C21—C17—C18	119.59 (19)
O23—Zn2—O11	105.43 (6)	C18—O18—Zn3	112.37 (13)
C9—Zn2—O12	117.31 (8)	O18—C18—C19	123.5 (2)
O23—Zn2—O12	92.80 (6)	O18—C18—C17	120.21 (18)
O11—Zn2—O12	78.77 (6)	C19—C18—C17	116.3 (2)
O24—Zn3—O23	79.77 (5)	C20—C19—C18	119.9 (2)
O24—Zn3—O4	103.06 (6)	C20—C19—H19	120.0
O23—Zn3—O4	97.63 (6)	C18—C19—H19	120.0
O24—Zn3—O3	162.57 (6)	C20—O20—C21	119.99 (18)
O23—Zn3—O3	82.81 (5)	C19—C20—O20	122.9 (2)
O4—Zn3—O3	79.64 (6)	C19—C20—H20	118.6
O24—Zn3—O18	89.61 (6)	O20—C20—H20	118.6
O23—Zn3—O18	168.68 (5)	C17—C21—O20	121.2 (2)
O4—Zn3—O18	88.54 (6)	C17—C21—C22	126.0 (2)
O3—Zn3—O18	107.74 (5)	O20—C21—C22	112.73 (19)
O24—Zn3—O17	102.00 (6)	C21—C22—H22A	109.5
O23—Zn3—O17	99.71 (5)	C21—C22—H22B	109.5
O4—Zn3—O17	151.62 (5)	H22A—C22—H22B	109.5
O3—Zn3—O17	80.44 (5)	C21—C22—H22C	109.5
O18—Zn3—O17	78.54 (5)	H22A—C22—H22C	109.5
O24—Zn3—Zn1	140.09 (4)	H22B—C22—H22C	109.5
O23—Zn3—Zn1	91.53 (4)	C23—O23—Zn2	135.97 (12)
O4—Zn3—Zn1	116.73 (4)	C23—O23—Zn3	111.61 (11)
O3—Zn3—Zn1	39.63 (4)	Zn2—O23—Zn3	112.17 (6)
O18—Zn3—Zn1	94.17 (4)	O23—C23—C27	125.00 (18)
O17—Zn3—Zn1	40.81 (4)	O23—C23—C24	115.78 (17)
C2—C1—Zn1	111.80 (15)	C27—C23—C24	119.19 (18)
C2—C1—H1A	109.3	C24—O24—Zn3	111.97 (12)
Zn1—C1—H1A	109.3	O24—C24—C25	123.28 (18)
C2—C1—H1B	109.3	O24—C24—C23	119.69 (17)
Zn1—C1—H1B	109.3	C25—C24—C23	117.02 (18)
H1A—C1—H1B	107.9	C26—C25—C24	119.46 (19)
C1—C2—H2A	109.5	C26—C25—H25	120.3
C1—C2—H2B	109.5	C24—C25—H25	120.3
H2A—C2—H2B	109.5	C26—O26—C27	120.69 (16)
C1—C2—H2C	109.5	O26—C26—C25	122.80 (19)
H2A—C2—H2C	109.5	O26—C26—H26	118.6
H2B—C2—H2C	109.5	C25—C26—H26	118.6
C3—O3—Zn1	140.03 (12)	C23—C27—O26	120.72 (17)
C3—O3—Zn3	110.75 (12)	C23—C27—C28	127.98 (18)
Zn1—O3—Zn3	98.92 (6)	O26—C27—C28	111.29 (16)
O3—C3—C7	123.35 (18)	C27—C28—H28A	109.5
O3—C3—C4	116.32 (17)	C27—C28—H28B	109.5
C7—C3—C4	120.32 (18)	H28A—C28—H28B	109.5
C4—O4—Zn3	112.23 (13)	C27—C28—H28C	109.5
O4—C4—C5	123.75 (19)	H28A—C28—H28C	109.5
O4—C4—C3	120.44 (18)	H28B—C28—H28C	109.5
C5—C4—C3	115.80 (19)	C30—C29—C34	117.7 (2)
C6—C5—C4	120.0 (2)	C30—C29—C35	121.2 (2)
C6—C5—H5	120.0	C34—C29—C35	121.1 (2)
C4—C5—H5	120.0	C31—C30—C29	121.7 (2)
C6—O6—C7	119.56 (17)	C31—C30—H30	119.1
C5—C6—O6	123.3 (2)	C29—C30—H30	119.1
C5—C6—H6	118.3	C30—C31—C32	120.1 (2)
O6—C6—H6	118.3	C30—C31—H31	120.0
C3—C7—O6	120.93 (19)	C32—C31—H31	120.0
C3—C7—C8	126.41 (19)	C31—C32—C33	119.4 (3)
O6—C7—C8	112.65 (18)	C31—C32—H32	120.3
C7—C8—H8A	109.5	C33—C32—H32	120.3
C7—C8—H8B	109.5	C34—C33—C32	120.7 (2)
H8A—C8—H8B	109.5	C34—C33—H33	119.6
C7—C8—H8C	109.5	C32—C33—H33	119.6
H8A—C8—H8C	109.5	C33—C34—C29	120.5 (2)
H8B—C8—H8C	109.5	C33—C34—H34	119.8
C10—C9—Zn2	116.61 (15)	C29—C34—H34	119.8
C10—C9—H9A	108.1	C29—C35—H35A	109.5
Zn2—C9—H9A	108.1	C29—C35—H35B	109.5
C10—C9—H9B	108.1	H35A—C35—H35B	109.5
Zn2—C9—H9B	108.1	C29—C35—H35C	109.5
H9A—C9—H9B	107.3	H35A—C35—H35C	109.5
C9—C10—H10A	109.5	H35B—C35—H35C	109.5
C9—C10—H10B	109.5	C41—C36—C37	118.2 (2)
H10A—C10—H10B	109.5	C41—C36—C42	121.0 (2)
C9—C10—H10C	109.5	C37—C36—C42	120.7 (2)
H10A—C10—H10C	109.5	C38—C37—C36	121.4 (2)
H10B—C10—H10C	109.5	C38—C37—H37	119.3
C11—O11—Zn1	129.03 (12)	C36—C37—H37	119.3
C11—O11—Zn2	112.67 (12)	C37—C38—C39	119.7 (2)
Zn1—O11—Zn2	109.73 (6)	C37—C38—H38	120.1
O11—C11—C15	124.91 (18)	C39—C38—H38	120.1
O11—C11—C12	115.63 (17)	C38—C39—C40	119.8 (2)
C15—C11—C12	119.34 (18)	C38—C39—H39	120.1
C12—O12—Zn2	112.83 (13)	C40—C39—H39	120.1
O12—C12—C13	123.14 (19)	C39—C40—C41	120.3 (2)
O12—C12—C11	120.04 (18)	C39—C40—H40	119.8
C13—C12—C11	116.82 (18)	C41—C40—H40	119.8
C14—C13—C12	119.68 (19)	C40—C41—C36	120.6 (2)
C14—C13—H13	120.2	C40—C41—H41	119.7
C12—C13—H13	120.2	C36—C41—H41	119.7
C14—O14—C15	120.55 (16)	C36—C42—H42A	109.5
O14—C14—C13	122.80 (19)	C36—C42—H42B	109.5
O14—C14—H14	118.6	H42A—C42—H42B	109.5
C13—C14—H14	118.6	C36—C42—H42C	109.5
C11—C15—O14	120.56 (18)	H42A—C42—H42C	109.5
C11—C15—C16	128.53 (19)	H42B—C42—H42C	109.5
O14—C15—C16	110.89 (17)		
			
C1—Zn1—Zn3—O24	114.71 (11)	C12—C11—C15—C16	−172.7 (2)
O11—Zn1—Zn3—O24	−68.86 (7)	C14—O14—C15—C11	−2.6 (3)
O3—Zn1—Zn3—O24	−152.61 (9)	C14—O14—C15—C16	176.04 (18)
O17—Zn1—Zn3—O24	27.30 (8)	C1—Zn1—O17—C17	−7.63 (16)
C1—Zn1—Zn3—O23	−169.47 (10)	O11—Zn1—O17—C17	−157.01 (13)
O11—Zn1—Zn3—O23	6.96 (5)	O3—Zn1—O17—C17	116.34 (14)
O3—Zn1—Zn3—O23	−76.78 (7)	Zn3—Zn1—O17—C17	116.27 (15)
O17—Zn1—Zn3—O23	103.12 (7)	C1—Zn1—O17—Zn3	−123.91 (8)
C1—Zn1—Zn3—O4	−70.14 (10)	O11—Zn1—O17—Zn3	86.71 (5)
O11—Zn1—Zn3—O4	106.30 (6)	O3—Zn1—O17—Zn3	0.06 (5)
O3—Zn1—Zn3—O4	22.55 (7)	O24—Zn3—O17—C17	76.61 (12)
O17—Zn1—Zn3—O4	−157.55 (7)	O23—Zn3—O17—C17	158.11 (12)
C1—Zn1—Zn3—O3	−92.69 (11)	O4—Zn3—O17—C17	−75.02 (16)
O11—Zn1—Zn3—O3	83.75 (7)	O3—Zn3—O17—C17	−120.96 (12)
O17—Zn1—Zn3—O3	179.91 (8)	O18—Zn3—O17—C17	−10.51 (12)
C1—Zn1—Zn3—O18	20.32 (10)	Zn1—Zn3—O17—C17	−120.90 (13)
O11—Zn1—Zn3—O18	−163.25 (6)	O24—Zn3—O17—Zn1	−162.49 (5)
O3—Zn1—Zn3—O18	113.01 (7)	O23—Zn3—O17—Zn1	−80.99 (6)
O17—Zn1—Zn3—O18	−67.09 (7)	O4—Zn3—O17—Zn1	45.88 (13)
C1—Zn1—Zn3—O17	87.41 (10)	O3—Zn3—O17—Zn1	−0.06 (5)
O11—Zn1—Zn3—O17	−96.16 (7)	O18—Zn3—O17—Zn1	110.39 (6)
O3—Zn1—Zn3—O17	−179.91 (8)	Zn1—O17—C17—C21	79.8 (2)
O11—Zn1—C1—C2	−154.88 (13)	Zn3—O17—C17—C21	−171.89 (16)
O3—Zn1—C1—C2	−31.49 (19)	Zn1—O17—C17—C18	−100.05 (17)
O17—Zn1—C1—C2	70.68 (17)	Zn3—O17—C17—C18	8.3 (2)
Zn3—Zn1—C1—C2	20.1 (2)	O24—Zn3—O18—C18	−90.51 (14)
C1—Zn1—O3—C3	−17.7 (2)	O23—Zn3—O18—C18	−70.3 (3)
O11—Zn1—O3—C3	125.88 (19)	O4—Zn3—O18—C18	166.41 (13)
O17—Zn1—O3—C3	−138.27 (19)	O3—Zn3—O18—C18	87.77 (14)
Zn3—Zn1—O3—C3	−138.2 (2)	O17—Zn3—O18—C18	11.82 (13)
C1—Zn1—O3—Zn3	120.50 (9)	Zn1—Zn3—O18—C18	49.72 (13)
O11—Zn1—O3—Zn3	−95.91 (6)	Zn3—O18—C18—C19	167.88 (16)
O17—Zn1—O3—Zn3	−0.06 (5)	Zn3—O18—C18—C17	−11.4 (2)
O24—Zn3—O3—C3	−107.5 (2)	O17—C17—C18—O18	2.0 (3)
O23—Zn3—O3—C3	−106.02 (12)	C21—C17—C18—O18	−177.82 (19)
O4—Zn3—O3—C3	−6.87 (12)	O17—C17—C18—C19	−177.37 (17)
O18—Zn3—O3—C3	78.21 (12)	C21—C17—C18—C19	2.8 (3)
O17—Zn3—O3—C3	152.82 (12)	O18—C18—C19—C20	−179.7 (2)
Zn1—Zn3—O3—C3	152.75 (15)	C17—C18—C19—C20	−0.3 (3)
O24—Zn3—O3—Zn1	99.71 (18)	C18—C19—C20—O20	−2.2 (3)
O23—Zn3—O3—Zn1	101.22 (6)	C21—O20—C20—C19	2.2 (3)
O4—Zn3—O3—Zn1	−159.62 (7)	O17—C17—C21—O20	177.28 (17)
O18—Zn3—O3—Zn1	−74.54 (6)	C18—C17—C21—O20	−2.9 (3)
O17—Zn3—O3—Zn1	0.06 (5)	O17—C17—C21—C22	−2.7 (3)
Zn1—O3—C3—C7	−38.8 (3)	C18—C17—C21—C22	177.1 (2)
Zn3—O3—C3—C7	−174.06 (16)	C20—O20—C21—C17	0.4 (3)
Zn1—O3—C3—C4	142.15 (16)	C20—O20—C21—C22	−179.60 (19)
Zn3—O3—C3—C4	6.9 (2)	C9—Zn2—O23—C23	−83.56 (19)
O24—Zn3—O4—C4	168.35 (13)	O11—Zn2—O23—C23	121.29 (17)
O23—Zn3—O4—C4	87.15 (14)	O12—Zn2—O23—C23	42.17 (18)
O3—Zn3—O4—C4	5.94 (13)	C9—Zn2—O23—Zn3	102.85 (10)
O18—Zn3—O4—C4	−102.39 (14)	O11—Zn2—O23—Zn3	−52.30 (8)
O17—Zn3—O4—C4	−40.14 (19)	O12—Zn2—O23—Zn3	−131.43 (7)
Zn1—Zn3—O4—C4	−8.46 (14)	O24—Zn3—O23—C23	−9.44 (12)
Zn3—O4—C4—C5	175.83 (16)	O4—Zn3—O23—C23	92.54 (13)
Zn3—O4—C4—C3	−4.2 (2)	O3—Zn3—O23—C23	171.02 (13)
O3—C3—C4—O4	−2.0 (3)	O18—Zn3—O23—C23	−30.0 (3)
C7—C3—C4—O4	178.91 (19)	O17—Zn3—O23—C23	−110.01 (12)
O3—C3—C4—C5	177.97 (17)	Zn1—Zn3—O23—C23	−150.23 (12)
C7—C3—C4—C5	−1.1 (3)	O24—Zn3—O23—Zn2	165.78 (8)
O4—C4—C5—C6	−179.1 (2)	O4—Zn3—O23—Zn2	−92.24 (7)
C3—C4—C5—C6	1.0 (3)	O3—Zn3—O23—Zn2	−13.76 (7)
C4—C5—C6—O6	0.0 (4)	O18—Zn3—O23—Zn2	145.3 (3)
C7—O6—C6—C5	−1.0 (3)	O17—Zn3—O23—Zn2	65.20 (7)
O3—C3—C7—O6	−178.77 (17)	Zn1—Zn3—O23—Zn2	24.98 (6)
C4—C3—C7—O6	0.2 (3)	Zn2—O23—C23—C27	17.4 (3)
O3—C3—C7—C8	0.3 (3)	Zn3—O23—C23—C27	−168.99 (16)
C4—C3—C7—C8	179.32 (19)	Zn2—O23—C23—C24	−164.84 (13)
C6—O6—C7—C3	0.8 (3)	Zn3—O23—C23—C24	8.8 (2)
C6—O6—C7—C8	−178.38 (19)	O23—Zn3—O24—C24	8.81 (13)
O23—Zn2—C9—C10	−6.7 (2)	O4—Zn3—O24—C24	−86.74 (14)
O11—Zn2—C9—C10	143.40 (14)	O3—Zn3—O24—C24	10.3 (3)
O12—Zn2—C9—C10	−120.88 (15)	O18—Zn3—O24—C24	−175.14 (14)
C1—Zn1—O11—C11	−73.88 (18)	O17—Zn3—O24—C24	106.67 (13)
O3—Zn1—O11—C11	150.99 (15)	Zn1—Zn3—O24—C24	88.82 (14)
O17—Zn1—O11—C11	67.18 (16)	Zn3—O24—C24—C25	172.62 (15)
Zn3—Zn1—O11—C11	109.78 (15)	Zn3—O24—C24—C23	−7.0 (2)
C1—Zn1—O11—Zn2	141.24 (10)	O23—C23—C24—O24	−1.3 (3)
O3—Zn1—O11—Zn2	6.11 (6)	C27—C23—C24—O24	176.61 (18)
O17—Zn1—O11—Zn2	−77.70 (7)	O23—C23—C24—C25	179.10 (17)
Zn3—Zn1—O11—Zn2	−35.10 (6)	C27—C23—C24—C25	−3.0 (3)
C9—Zn2—O11—C11	113.57 (14)	O24—C24—C25—C26	−179.2 (2)
O23—Zn2—O11—C11	−91.92 (13)	C23—C24—C25—C26	0.4 (3)
O12—Zn2—O11—C11	−2.10 (12)	C27—O26—C26—C25	−0.3 (3)
C9—Zn2—O11—Zn1	−95.40 (10)	C24—C25—C26—O26	1.3 (3)
O23—Zn2—O11—Zn1	59.12 (7)	O23—C23—C27—O26	−178.22 (17)
O12—Zn2—O11—Zn1	148.94 (7)	C24—C23—C27—O26	4.1 (3)
Zn1—O11—C11—C15	42.4 (3)	O23—C23—C27—C28	2.7 (3)
Zn2—O11—C11—C15	−173.51 (16)	C24—C23—C27—C28	−174.98 (19)
Zn1—O11—C11—C12	−141.61 (14)	C26—O26—C27—C23	−2.5 (3)
Zn2—O11—C11—C12	2.5 (2)	C26—O26—C27—C28	176.74 (17)
C9—Zn2—O12—C12	−122.96 (14)	C34—C29—C30—C31	0.0 (3)
O23—Zn2—O12—C12	106.61 (14)	C35—C29—C30—C31	178.3 (2)
O11—Zn2—O12—C12	1.42 (13)	C29—C30—C31—C32	0.5 (4)
Zn2—O12—C12—C13	179.17 (15)	C30—C31—C32—C33	−0.3 (4)
Zn2—O12—C12—C11	−0.6 (2)	C31—C32—C33—C34	−0.5 (4)
O11—C11—C12—O12	−1.3 (3)	C32—C33—C34—C29	1.0 (4)
C15—C11—C12—O12	174.89 (18)	C30—C29—C34—C33	−0.8 (3)
O11—C11—C12—C13	178.94 (17)	C35—C29—C34—C33	−179.0 (2)
C15—C11—C12—C13	−4.9 (3)	C41—C36—C37—C38	−1.1 (3)
O12—C12—C13—C14	−178.7 (2)	C42—C36—C37—C38	177.4 (2)
C11—C12—C13—C14	1.1 (3)	C36—C37—C38—C39	0.3 (3)
C15—O14—C14—C13	−1.4 (3)	C37—C38—C39—C40	0.5 (3)
C12—C13—C14—O14	2.1 (3)	C38—C39—C40—C41	−0.5 (3)
O11—C11—C15—O14	−178.51 (17)	C39—C40—C41—C36	−0.3 (3)
C12—C11—C15—O14	5.7 (3)	C37—C36—C41—C40	1.1 (3)
O11—C11—C15—C16	3.1 (3)	C42—C36—C41—C40	−177.4 (2)

### Hydrogen-bond geometry (Å, º)

Please define Cg1 and Cg2

**Table:**

*D*—H···*A*	*D*—H	H···*A*	*D*···*A*	*D*—H···*A*
C6—H6···*Cg*1^i^	0.95	2.53	3.477 (3)	172
C14—H14···O24^ii^	0.95	2.39	3.286 (3)	156
C20—H20···O17^iii^	0.95	2.40	3.332 (3)	166
C26—H26···O18^iv^	0.95	2.33	3.167 (3)	146
C28—H28*B*···*Cg*2^v^	0.98	2.72	3.411 (3)	128

Symmetry codes: (i) −*x*, −*y*+1, −*z*; (ii) −*x*+1/2, *y*+1/2, −*z*+1/2; (iii) −*x*+1/2, *y*−1/2, −*z*+1/2; (iv) −*x*−1/2, *y*+1/2, −*z*+1/2; (v) *x*−1, *y*+1, *z*.
